# Transcriptome sequencing and validation of potential biomarkers associated with cognitive impairment after hypertensive intracerebral hemorrhage

**DOI:** 10.3389/fmolb.2026.1877108

**Published:** 2026-07-29

**Authors:** Yao Tang, Boyin Yang, Xingmei Luo

**Affiliations:** 1 Department of General Ward, Affiliated Hospital of Guizhou Medical University, Guiyang, China; 2 Physical Examination Center, Affiliated Hospital of Guizhou Medical University, Guiyang, China

**Keywords:** biomarkers, cognitive impairment, hypertensive intracerebral hemorrhage, RT-qPCR, transcriptome sequencing analysis

## Abstract

**Background:**

Hypertensive cerebral hemorrhage (HCH) accounts for the majority of spontaneous intracerebral hemorrhage (ICH) cases. Cognitive impairment (CI) is a major contributor to long-term disability following ICH, yet the molecular mechanisms underlying CI in hypertensive ICH remain poorly understood. Therefore, identifying biomarkers capable of effectively diagnosing or predicting CI in HCH is particularly critical for managing patients with hypertensive cerebral hemorrhage with cognitive impairment (HCHwCI).

**Methods:**

In this research, clinical samples underwent transcriptome sequencing analysis. Identification of biomarkers associated with CI in HCH was achieved through differential expression analysis, protein-protein interaction (PPI) network construction, and expression level evaluation. Subsequently, to investigate the molecular mechanisms of the biomarkers in HCHwCI, comprehensive analyses were performed, including gene set enrichment analysis (GSEA), molecular regulatory network construction, drug prediction, and molecular docking. Finally, the expression of biomarkers was detected by reverse transcription-quantitative polymerase chain reaction (RT-qPCR).

**Results:**

Using 247 differentially expressed CI-related genes (CRGs), we identified CXCL8 and FCGR3B as significantly upregulated biomarkers in HCH patients with CI, a finding subsequently validated by RT-qPCR. CXCL8 and FCGR3B were also found to be significantly enriched in oxidative phosphorylation and other related pathways. The molecular regulation study found that 67 microRNAs (miRNAs) were predicted to regulate CXCL8, and 58 miRNAs were predicted to regulate FCGR3B. Among them, hsa-miR-3168 and hsa-miR-567 jointly regulated both CXCL8 and FCGR3B. In addition, there were 29 transcription factors (TFs) were predicted to target CXCL8, and there were 2 TFs were predicted to target FCGR3B, such as the pairs of CXCL8-STAT6 and FCGR3B-YY1. Moreover, through drug prediction, there were 542 drugs targeting CXCL8 and FCGR3B (such as methotrexate and hydrogen peroxide). Among them, the | Total Score | of FCGR3B and clozapine is 6.3 kcal/mol, and the | Total Score | of CXCL8 and 6401-97-4 is 5.1 kcal/mol, both indicating good binding activity *in silico*, serving as a proof-of-concept for the computational docking approach.

**Conclusion:**

In this study, two potential biomarkers, CXCL8 and FCGR3B, were preliminarily identified through transcriptome sequencing combined with biological validation methods to provide novel insights into future therapeutic strategies and research directions for HCHwCI.

## 1 Introduction

Intracerebral hemorrhage (ICH) is a serious neurological disease with high mortality, accounting for 10%–15% of all stroke subtypes (de Oliveira Manoel, 2020). This condition has a sudden onset and progresses rapidly, with up to 70% of cases associated with a history of hypertension (Zille et al., 2022). Long term hypertension not only directly damages the cerebral vascular endothelium, increases vascular fragility, induces vascular rupture, hematoma, and neurological damage, but also exacerbates target organ damage through immune inflammatory mechanisms (immune cell activation, pro-inflammatory cytokine release, etc.), ultimately leading to cognitive impairment, affecting memory, attention, and executive function, and severely reducing patient independence (Pasi et al., 2021; Guzik et al., 2024). At present, the methods for treating cerebral hemorrhage include blood pressure management, surgical intervention, and coagulation reversal (Magid-Bernstein et al., 2022; Aydin and Peker, 2025). Therapeutic options for cognitive decline after hypertensive intracerebral hemorrhage are still constrained, and specific biomarkers for early detection and risk assessment are lacking. Therefore, identifying biomarkers that can effectively identify CI in HCH is particularly important for the treatment of HCHwCI.

RNA sequencing (RNA-Seq) enables comprehensive transcriptome analysis and has proven invaluable in identifying disease biomarkers and therapeutic targets. In neuroscience, RNA-Seq technology has been effectively employed to examine transcriptional alterations in neurological conditions like Alzheimer’s disease and stroke, greatly enhancing the identification of disease biomarkers and therapeutic target screening (Mathys et al., 2019). For instance, Peripheral blood RNA-Seq in intracerebral hemorrhage patients demonstrated the pivotal involvement of inflammatory responses, oxidative stress, and vascular dysfunction in disease development (Sang et al., 2017). Although no direct transcriptome sequencing studies on human HCHwCI have been reported to date, an RNA-Seq study in hypertensive mice demonstrated that the combination of hypertension and stroke induces abnormal changes in differentially expressed genes associated with neuroinflammation in the brain. These molecular-level alterations were directly correlated with worsening cognitive impairment (Wong Zhang et al., 2024), demonstrating that RNA-Seq can reveal key molecular pathways and pathological processes underlying cognitive dysfunction (Swe et al., 2018). This holds significant implications for screening biomarkers for HCHwCI, elucidating disease mechanisms, and guiding therapeutic strategies.

This study identified biomarkers associated with CI in HCH based on transcriptome sequencing data from clinical patients, through differential expression analysis, PPI screening, and expression validation. Furthermore, gene set enrichment analysis, subcellular localization, chromosomal mapping, and GeneMANIA analysis were performed based on these biomarkers. To investigate underlying mechanisms and therapeutic implications, regulatory network analysis and drug target prediction were conducted. Finally, biomarkers were validated via reverse transcription-quantitative polymerase chain reaction (RT-qPCR) to assess key gene expression.

## 2 Methods

### 2.1 Sample collection and sequencing

Peripheral blood samples were collected from three cohorts at The Affiliated Hospital of Guizhou Medical University: 3 patients with hypertension (HTN), 3 with hypertensive cerebral hemorrhage without cognitive impairment (HCHsCI), and 3 with HCHwCI. Cognitive assessment was performed by trained neurologists using the Montreal Cognitive Assessment (MoCA) scale. Cognitive impairment was defined as a MoCA score <26, with one point added for participants with ≤12 years of education. Assessment was conducted within 7 days after symptom onset (for HCH patients) or during routine outpatient visits (for HTN controls). Patients with pre-existing cognitive impairment (e.g., Alzheimer’s disease, vascular dementia), severe aphasia, or inability to complete the assessment were excluded. For the HCHsCI group, MoCA scores were ≥26, confirming the absence of cognitive impairment. With ethics approval (Approval No. [895]) and informed consent, peripheral blood mononuclear cells (PBMCs) were isolated by Ficoll-Paque PLUS (GE Healthcare) centrifugation. RNA extraction (TRIzol Reagent, Invitrogen) was followed by DNase treatment (RQ1 DNase, Promega) and quality assessment. Strand-specific sequencing libraries were generated from 1 µg total RNA using VAHTS mRNA Capture Beads (Vazyme) for Poly(A)+ selection and the VAHTS Universal V8 RNA-seq Library Prep Kit for Illumina (Vazyme) with VAHTS RNA Multiplex Oligos Set 1 (Vazyme) for library construction. Libraries were quality-controlled (Agilent 2100 Bioanalyzer) and sequenced on an Illumina NovaSeq 6000 system for 150 bp paired-end reads.

### 2.2 Quality control of transcriptomic data

The sequences obtained by sequencing are generally called raw sequences (raw reads), which are stored in the FastQC software (v 0.11.9) format and contain unqualified reads, such as sequencing primers, and low-quality reads at the end of sequence reads. In order to obtain clean reads for data analysis, the low-quality reads were filtered using FastQC software. Then, the base quality value formula in Phred software was used to analyze the sequencing sample data and calculate the base error rate. The higher the base quality value, the more reliable and accurate the base identification. The separation of A-T and C-G was detected through a base type distribution check. The Ensembl database (https://asia.ensembl.org/info/about/species.html) served as the source for downloading the reference human genome and its annotation file. Hisat2 was used to map the human clean reads in this study to the reference sequence. According to the mapping results, the transcripts were assembled using the Cufflinks (v 2.2.1) program to obtain the final count data.

### 2.3 Differential expression analysis

For the identification of differentially expressed genes, the unnormalized counts matrix from the sequencing dataset was utilized as the input. The DESeq2 package (v 3.58.1) (Love et al., 2014) was applied to perform the differential expression analysis. The dataset comprised three groups: hypertensive controls (Control, n = 3), hypertensive cerebral hemorrhage without cognitive impairment (HCHsCI, n = 3), and hypertensive cerebral hemorrhage with cognitive impairment (HCHwCI, n = 3). Three specific comparisons were conducted: (1) DEGs1 (HCHsCI vs. Control), (2) DEGs2 (HCHwCI vs. Control), and (3) DEGs3 (HCHwCI vs. HCHsCI). The thresholds for defining significant differential expression across all comparisons were set at |log_2_FoldChange (FC)| > 0.5 and an unadjusted p-value <0.05. Given the complex and heterogeneous pathological nature of cognitive impairment following intracerebral hemorrhage, and considering the limited sample size of this exploratory study, a relatively permissive threshold (|log_2_FC| > 0.5 and unadjusted p-value <0.05) was applied. This approach was strategically chosen to prevent the omission of biologically meaningful genes that exhibit subtle but coordinated expression changes, a phenomenon frequently observed in transcriptomic studies of neurological disorders. To mitigate the risk of false positives inherent to this relaxed threshold, downstream experimental validation via RT-qPCR was strictly implemented for the key identified hub genes. To visualize the global distribution of DEGs1, DEGs2, and DEGs3, volcano plots and heatmaps came into being through the use of the ggplot2 (v 3.4.1) (Ito and Murphy, 2013) and ComplexHeatmap package (v 2.14.0) (Gu et al., 2016).

### 2.4 Identification of candidate genes

The intersections among DEGs1, DEGs2, and DEGs3 were analyzed using the VennDiagram package (v 1.7.3) (Hu et al., 2024) to obtain the genes related to CI (CI-related genes, CRGs) in HCH, and mark them as differential CRGs. The clusterProfiler package (v 4.2.2) (Huang et al., 2024) was employed for Gene Ontology (GO) and Kyoto Encyclopedia of Genes and Genomes (KEGG) enrichment analyses of the differential CRGs, with a significance threshold of p < 0.05. The GO system categorizes genes into 3 functional aspects: molecular function (MF), cellular component (CC), and biological process (BP). The KEGG database was utilized to annotate identified genes and analyze their involvement in key metabolic pathways and signal transduction mechanisms. Differential CRGs were submitted to the Search Tool for the Retrieval of Interacting Genes/Proteins (STRING) database (https://string-db.org/) to construct a protein-protein interaction (PPI) network with a confidence score >0.4, a standard threshold recommended by the database to optimally balance data coverage and false positive interactions. Cytoscape Software (v 3.10.2) (Geng et al., 2024) was used for network visualization. To ensure statistical robustness and topological importance within the network, particularly in the absence of an external transcriptomic validation cohort, three distinct centrality algorithms (Degree, Maximal Clique Centrality [MCC], and Edge Percolated Component [EPC]) from the CytoHubba plugin were utilized to score the differential CRGs. Candidate hub genes were subsequently identified by intersecting the top 3 genes from each algorithm, a consensus statistical approach widely recognized for reliably isolating true biological drivers while minimizing method-specific biases.

### 2.5 Identification of biomarkers

The Wilcoxon test (P < 0.05) was applied to evaluate expression differences of candidate genes between HCHwCI and HCHsCI groups for further biomarker identification. Genes showing significant differential expression between these two groups were subsequently designated as biomarkers.

### 2.6 Gene set enrichment analysis (GSEA)

The “c2.cp.kegg.v7.5.1.symbols.gmt” gene set from Molecular Signatures Database (MSigDB) (https://www.gsea-msigdb.org/gsea/msigdb) was selected to explore biomarker-related functions and pathways in disease development. Spearman correlations between biomarkers and all genes in HCHwCI and HCHsCI groups were computed with the psych package (v 2.2.9) (Silva-Martins et al., 2023), followed by gene ranking based on correlation coefficients. GSEA using clusterProfiler package (v 4.2.2) (Huang et al., 2024) (p < 0.05, |normalized enrichment score (NES)| > 1) identified significant pathways, and the top five (p < 0.05) were plotted with enrichplot package (v 1.18.0) (Zhang et al., 2019).

### 2.7 Subcellular and chromosomal localization of biomarkers

To further investigate biomarker functions, FASTA files of biomarkers were first retrieved from the NCBI database (https://www.ncbi.nlm.nih.gov/). The mRNALocator database (http://bio-bigdata.cn/mRNALocater/) was employed for subcellular localization predictions, with outcomes displayed as stacked bar plots using ggplot2 (v 3.4.1) (Cao et al., 2023).

In order to explore the localization of biomarkers on human chromosomes, the human cytogenetic banding data (UCSC HG19 Human CytoBandIdeogram dataset (https://hgdownload.cse.ucsc.edu/goldenpath/hg19/bigZips/)) were downloaded. These data were processed and analyzed using the RCircos package (v 1.2.2) (Xie et al., 2024) to generate a circos plot, thereby visualizing the distribution patterns of biomarkers across human chromosomes.

### 2.8 GeneMANIA analysis

Biomarker genes were input into the GeneMANIA database (https://genemania.org/) to identify functionally related genes, which were then screened based on functional similarity.

### 2.9 Molecular regulatory network analysis

To explore the regulatory interaction between biomarkers and miRNAs, the miRDB database (https://www.mirdb.org/) was utilized to predict the miRNA targets of biomarkers. Subsequently, Cytoscape software (v 3.10.2) was used to visualize the predicted biomarker-miRNA interaction network. Potential transcription factors (TFs) regulating the biomarkers were predicted via the TRRUST database (https://www.grnpedia.org/trrust/) on the NetworkAnalyst online platform (https://www.networkanalyst.ca/) to explore TF-mediated transcriptional regulation. The network was subsequently visualized using Cytoscape software (v 3.10.2).

### 2.10 Drug predictive and molecular docking analysis

The Drug Signature Database (DSigDB, https://www.dsigdb.org/) was employed to predict drug candidates associated with HCHwCI biomarkers (p < 0.05) for potential therapeutic targeting. The visualization of the drug-biomarker interaction network was carried out by means of Cytoscape software (v 3.10.2) (Geng et al., 2024). Drugs with the highest correlation scores were selected as top candidates. To further verify the binding ability of the targeted drugs to the biomarkers, drugs with the highest scores related to the biomarkers and with an absolute value of the Total Score greater than 0.5 (namely, 6401-97-4 and clozapine) were selected for molecular docking. The 3D protein structures of the key biomarker targets (CXCL8 and FCGR3B) were downloaded from the PDB database (https://www.rcsb.org), and the 3D structures of the drug ligands were retrieved from the PubChem database (https://pubchem.ncbi.nlm.nih.gov/). The protein and ligand files were subsequently uploaded to the CB-Dock2 web server (https://cadd.labshare.cn/cb-dock2/php/index.php). The CB-Dock2 platform was utilized to perform a blind docking approach. During this automated process, the server intrinsically executed essential preprocessing steps for both the proteins and drug molecules, including the removal of water molecules and the addition of hydrogen atoms. Finally, the docking simulations were completed and the binding free energies were calculated.

### 2.11 RT-qPCR validation of biomarkers

Clinical validation of biomarker expression against bioinformatics predictions was conducted using blood samples from 10 patients (3 HTN, 3 HCHsCI, 4 HCHwCI) at the Affiliated Hospital of Guizhou Medical University. Written informed consent was obtained, and ethical approval was granted by the hospital’s Ethics Committee (Approval No. [895]). Using GraphPad Prism 10 for visualization and statistics, total RNA was extracted (Trizol, Vazyme Biotech Co., Nanjing, China) and reverse-transcribed (Hifair® III 1st Strand cDNA Synthesis SuperMix for qPCR, Yysen Biotechnology, Shanghai, China). RT-qPCR was run with 2 × Universal Blue SYBR Green qPCR Master Mix (Servicebio, Wuhan, China) and GAPDH reference. Primers are in Supplementary Data Sheet S1. Expression levels were calculated via the 2^−ΔΔCt^ method and statistical significance was determined using the unpaired Student’s t-test (P < 0.05).

### 2.12 Statistical analysis

Statistical analyses and data visualizations were performed using R software (v 4.2.2) and GraphPad Prism 10. For the bioinformatics analysis, the nonparametric Wilcoxon rank-sum test was employed to assess the differential expression of candidate genes between two respective sample groups. For the experimental validation (RT-qPCR), continuous variables were presented as the mean ± standard error of the mean (SEM). Differences between two independent experimental groups were evaluated using the unpaired Student’s t-test, assuming a normal distribution. A two-sided p-value < 0.05 was considered statistically significant.

Important statistical note: Given the critically small sample sizes in the present study (n = 3 or 4 per group), testing for normality and homogeneity of variance lacks sufficient power. Consequently, the statistical robustness is inherently limited, which represents a major limitation of this exploratory study.

## 3 Results

### 3.1 Baseline characteristics of patients

The baseline clinical characteristics of both the transcriptomic sequencing cohort (n = 9) and the independent RT-qPCR validation cohort (n = 10) are summarized in Table 1. Demographic data, including age, sex, education level, and underlying conditions, showed no significant differences among the three groups within each cohort (p > 0.05), indicating excellent comparability. As expected, the MoCA scores were significantly lower in the HCHwCI groups compared to the HTN and HCHsCI groups in both cohorts (P < 0.01).

**TABLE 1:** Baseline characteristics of the sequencing and validation cohorts.

Variable	Transcriptomic sequencing cohort (n = 9)				RT-qPCR validation cohort (n = 10)			
	HTN (n = 3)	HCHsCI (n = 3)	HCHwCI (n = 3)	P-value	HTN (n = 3)	HCHsCI (n = 3)	HCHwCI (n = 4)	P-value
Age, years	61.00 ± 10.54	64.33 ± 11.06	66.67 ± 11.68	0.76	56.67 ± 7.77	76.67 ± 22.68	75.00 ± 5.94	0.17
Sex, n (%)				1				1
Male	1 (33.33)	2 (66.67)	2 (66.67)		1 (33.33)	2 (66.67)	2 (50.00)	
Female	2 (66.67)	1 (33.33)	1 (33.33)		2 (66.67)	1 (33.33)	2 (50.00)	
Education, years	8.33 ± 2.52	7.33 ± 2.52	6.67 ± 2.08	0.63	8.67 ± 2.08	6.67 ± 5.69	6.25 ± 2.63	0.52
Hypertension duration, years	5.00 ± 3.61	3.67 ± 3.06	4.00 ± 2.00	0.78	8.00 ± 2.00	11.00 ± 8.54	11.50 ± 5.69	0.67
MoCA score	27.00 ± 1.00	26.67 ± 0.58	20.33 ± 1.53	<0.01	26.00 ± 1.00	22.00 ± 1.00	20.50 ± 1.29	<0.01
Diabetes mellitus, n (%)	1 (33.33)	1 (33.33)	2 (66.67)	0.64	1 (33.33)	1 (33.33)	2 (50.00)	0.88
Smoking history, n (%)	1 (33.33)	2 (66.67)	1 (33.33)	0.64	1 (33.33)	1 (33.33)	1 (25.00)	0.79

Data are presented as mean ± standard deviation (SD) for continuous variables and n (%) for categorical variables. HTN, hypertension; HCHsCI, hypertensive cerebral hemorrhage without cognitive impairment; HCHwCI, hypertensive cerebral hemorrhage with cognitive impairment; MoCA, montreal cognitive assessment.

### 3.2 High-quality of transcriptome data

**FIGURE 1:**
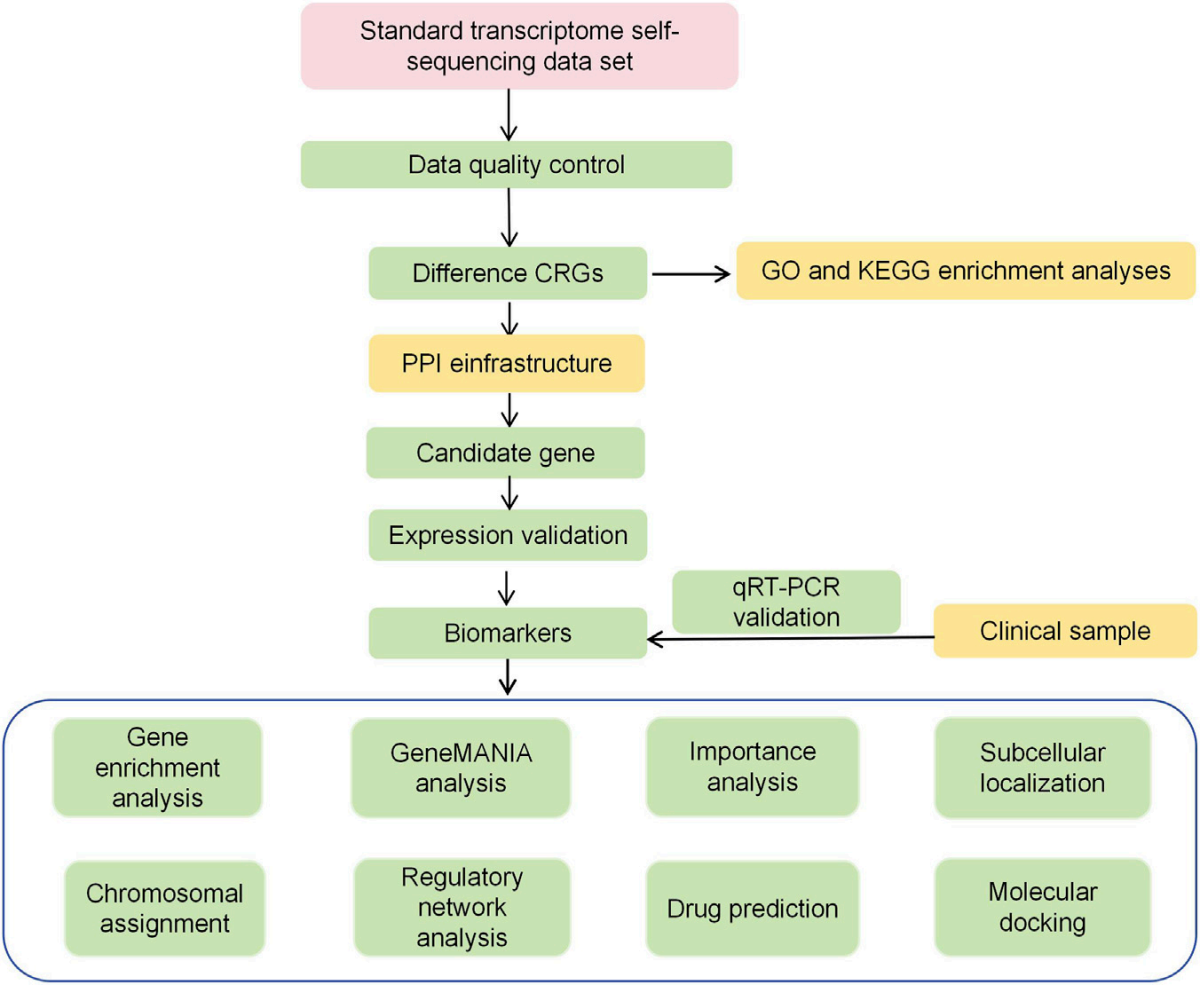
Flow chart of the study. Transcriptome data were quality-controlled, followed by screening of candidate regulatory genes (CRGs), functional enrichment, network analysis, drug and biomarker prediction. Candidate genes were analyzed by molecular docking and validated via qRT-PCR using clinical samples.

**FIGURE 2:**
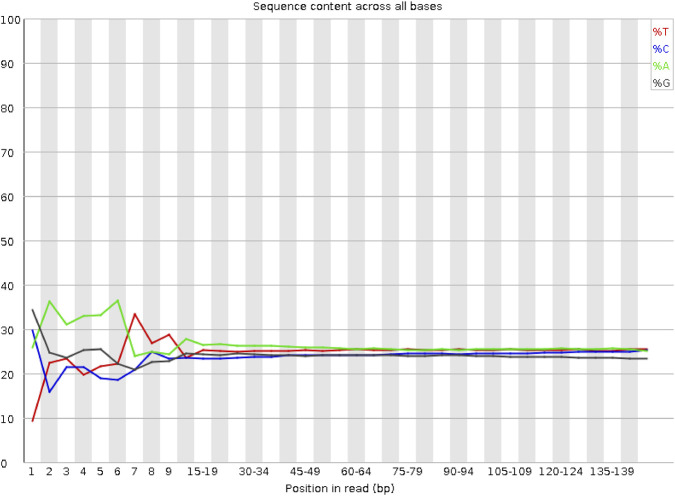
Single-base quality distribution plot of sample CI_1 (Control group). No separation was observed in A/T and C/G content, which remained stable overall, indicating no significant base bias in the sequencing library.

The overall study design is shown in Figure 1. In this study, the quality of raw transcriptomic sequencing data was rigorously evaluated (Table 2). Q20 and Q30 scores for all samples were calculated to exceed 97%, with GC content consistently maintained within the optimal range of 40%–50% (Supplementary Data Sheet S2), demonstrating compliance with downstream analytical requirements. Additionally, ridgeline plot profiling exhibited congruent expression density distributions among samples, with the majority of transcripts concentrated within a defined FPKM range, suggesting conserved expression hierarchies (Figure 2). Collectively, these quality metrics confirmed the reliability of the transcriptome sequencing data for downstream bioinformatic analysis.

**TABLE 2:** Alignment rate results.

Sample	Total_reads	Mapped (%)
CI_1_Clean_Data	78675696	97.31
CI_2_Clean_Data	63183108	96.81
CI_3_Clean_Data	75719116	96.59
H_1_Clean_Data	59529990	96.5
H_2_Clean_Data	70679314	96.51
H_3_Clean_Data	67271264	96.86
NCI_1_Clean_Data	72598662	97.24
NCI_2_Clean_Data	62624770	97.04
NCI_3_Clean_Data	73982076	96.85

### 3.3 Identification of DEGs1, DEGs2, DEGs3

**FIGURE 3:**
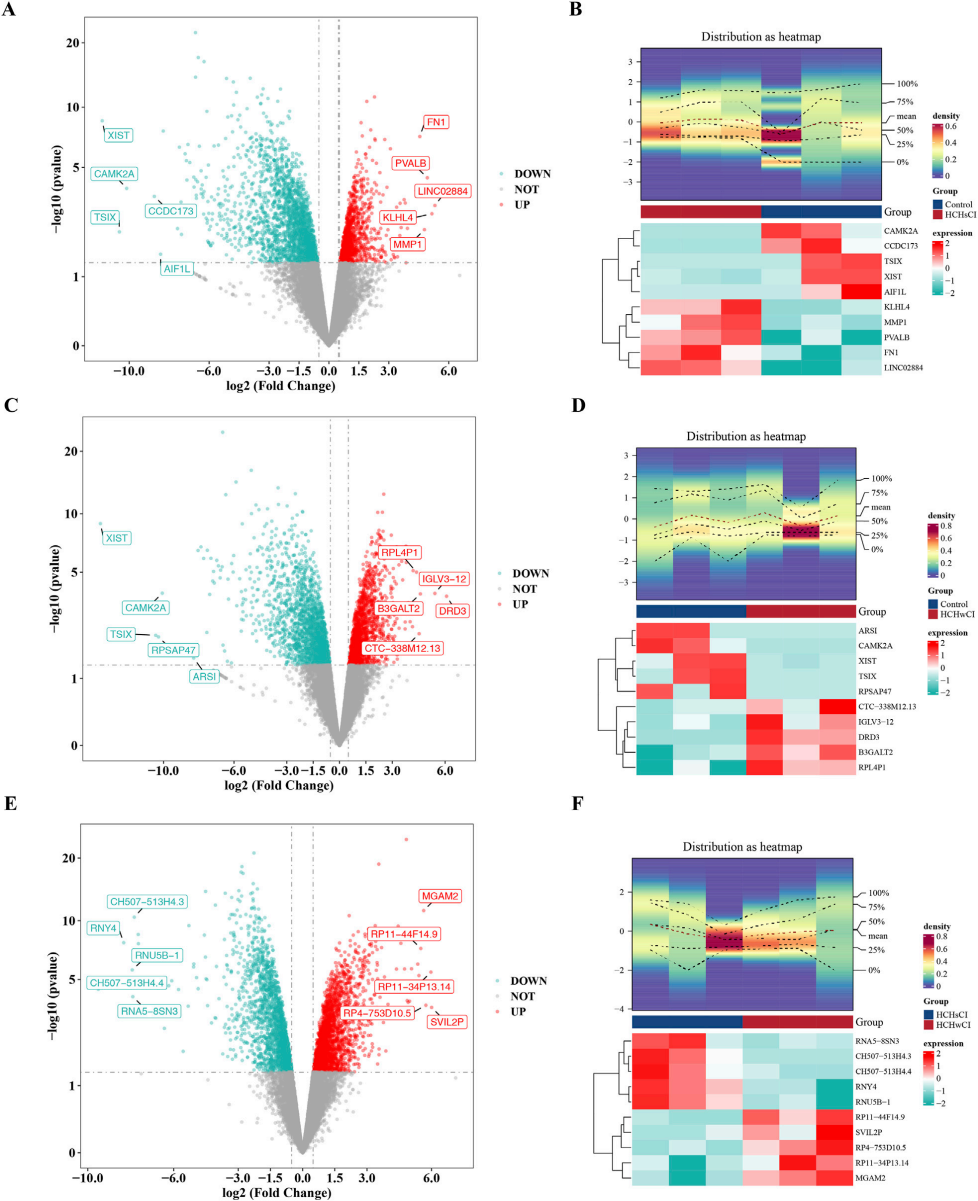
Volcano plots and heatmaps of DEGs. **(A)** Volcano plot of DEGs between the HCHsCI group and the hypertensive control group (Control). Red dots represent 3,694 upregulated DEGs, while green dots represent 2,459 downregulated DEGs. The top 5 most significantly up- and downregulated genes by fold change are labeled. **(B)** Expression heatmap of DEGs between the HCHsCI group and the Control group. Red boxes indicate upregulated DEGs; green boxes indicate downregulated DEGs. **(C)** Volcano plot of DEGs between HCHwCI and Control groups. Red dots: 3,815 upregulated DEGs; green dots: 2,133 downregulated DEGs. The top 5 genes with the highest fold changes are labeled. **(D)** Heatmap of differentially expressed genes between HCHwCI and Control groups. **(E)** Volcano plot of DEGs between the HCHwCI group and the HCHsCI group. Red dots represent 4,736 upregulated DEGs; green dots represent 2,373 downregulated DEGs. The top 5 genes with the highest fold changes are labeled in the figure. **(F)** Expression heatmap of DEGs between the HCHwCI group and the HCHsCI group.

In the transcriptome sequencing data of HCHsCI and control samples (Control), a total of 3,694 DEGs were identified (HCHsCI vs. Control). Among these DEGs1, 1,235 were upregulated in HCHsCI samples, while 2,459 were downregulated (Supplementary Data Sheet S3). In the comparison between HCHwCI and hypertensive Control samples, a total of 3,815 DEGs2 (HCHwCI vs. Control) were detected. Among DEGs2, 1,682 showed increased expression while 2,133 were decreased in HCHwCI samples (Supplementary Data Sheet S4). In the comparison of HCHwCI and HCHsCI samples (HCHwCI vs. HCHsCI), a number of DEGs3 were identified. Specifically, a total of 4,736 genes were identified, with 2,363 upregulated and 2,373 downregulated in HCHwCI samples (Supplementary Data Sheet S5). The five genes with the greatest |log2 FC| among DEGs1, DEGs2, and DEGs3 were shown for both up- and downregulation (Figures 3A–F).

### 3.4 Identification 247 differential CRGs

**FIGURE 4:**
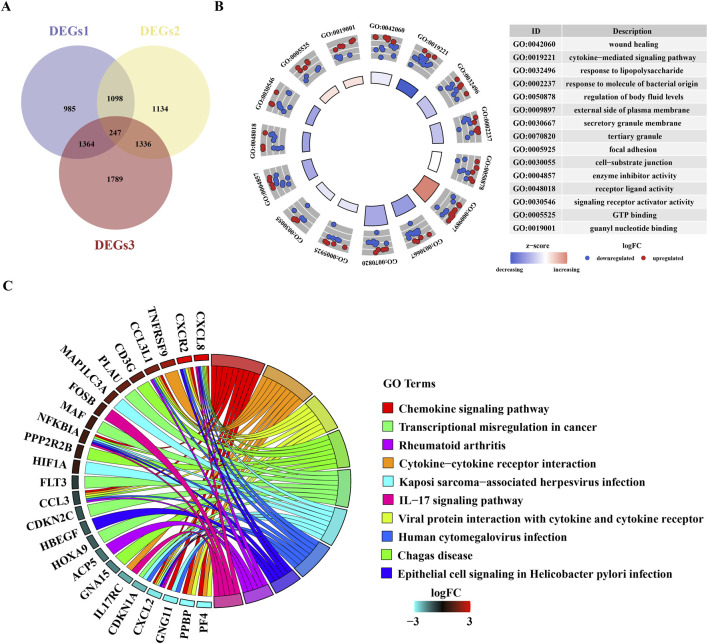
Identification and enrichment analysis of common genes. **(A)** Venn diagram showing the identification of 247 common differentially expressed genes (CRGs) between HCH and CI datasets using WGCNA. **(B)** GO functional enrichment analysis of the 247 CRGs, categorized into Biological Process (BP, 336 terms), Cellular Component (CC, 41 terms), and Molecular Function (MF, 55 terms). **(C)** KEGG pathway enrichment analysis. The top 10 pathways ranked by the number of enriched genes are presented (23 pathways identified in total).

DEGs1, DEGs2, and DEGs3 were intersected to obtain CRGs from 247 HCH samples, and these CRGs were recorded as differential CRGs (Figure 4A). GO enrichment analysis was then conducted on these differential CRGs, classifying them into 336 BPs, 41 CCs, and 55 MFs (Supplementary Data Sheet S6). In the biological process category, DEGs were significantly enriched in wound healing (GO:0042060). In the cellular component category, the external side of the plasma membrane (GO:0009897) was significantly enriched, and in the molecular function category, enzyme inhibitor activity (GO:0004857) was significantly enriched (p < 0.05) (Figure 4B). Finally, KEGG pathway analysis revealed that 23 key biological processes, including the Chemokine signaling pathway, Transcriptional misregulation in cancer, Rheumatoid arthritis, and the interaction between cytokines and their receptors, were significantly enriched (p < 0.05) (Figure 4C, Supplementary Data Sheet S7).

### 3.5 Acquisition of 2 biomarkers

**FIGURE 5:**
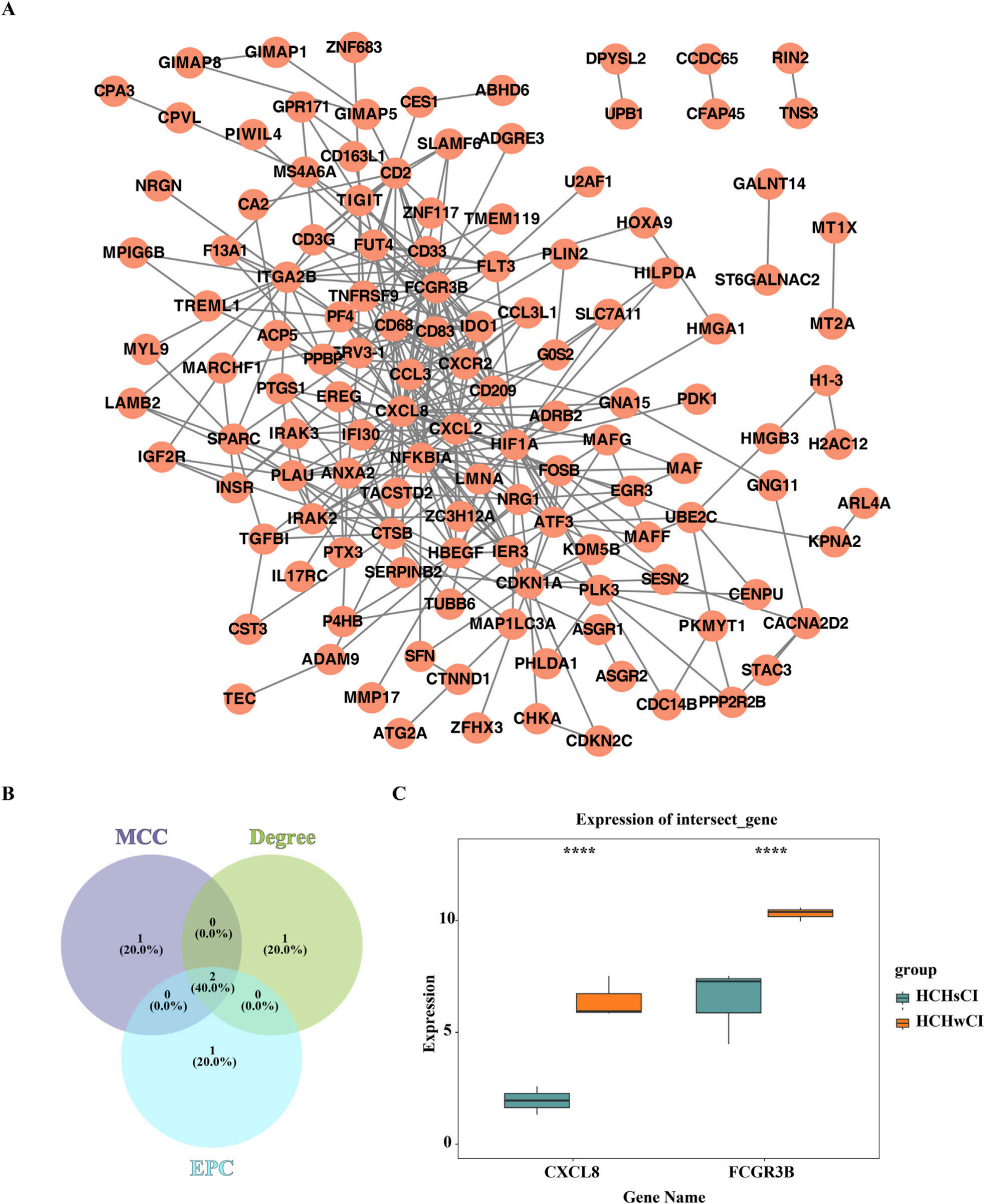
Protein-protein interactions (PPIs) of DEGs and identification of hub genes. **(A)** Protein-protein interaction network of differentially expressed genes constructed via the STRING platform, comprising 128 hub genes and 346 edges. Red nodes represent upregulated differentially expressed genes, while blue nodes denote downregulated differentially expressed genes. **(B)** Protein-protein interaction hub genes identified using three distinct algorithms (Degree, EPC, MCC). **(C)** Expression analysis of hub genes between HCHwCI and HCHsCI groups, compared using the Wilcoxon signed-rank test.

To gain a deeper understanding of protein-protein interactions encoded by 247 differential CRGs, a PPI network (interaction score >0.4) was constructed. Among them, 119 genes had no interaction with other genes. There were 346 interactions between the remaining genes. These interactions included between CD33 and FCGR3B, as well as between CXCL8 and CXCL3, etc., (Figure 5A). The top 3 genes were identified by three different algorithms (Degree, MCC, and EPC), and two candidate genes, CXCL8 and FCGR3B, were finally selected through intersection analysis (Figure 5B, Supplementary Data Sheets S8–S10). Expression levels of CXCL8 and FCGR3B differed significantly between HCHwCI and HCHsCI samples according to the Wilcoxon test (P < 0.05) applied to the transcriptome sequencing data. Meanwhile, CXCL8 and FCGR3B were significantly upregulated in HCHwCI samples (Figure 5C). Therefore, CXCL8 and FCGR3B were selected as biomarkers for subsequent analysis.

### 3.6 Enrichment analysis of 2 biomarkers

**FIGURE 6:**
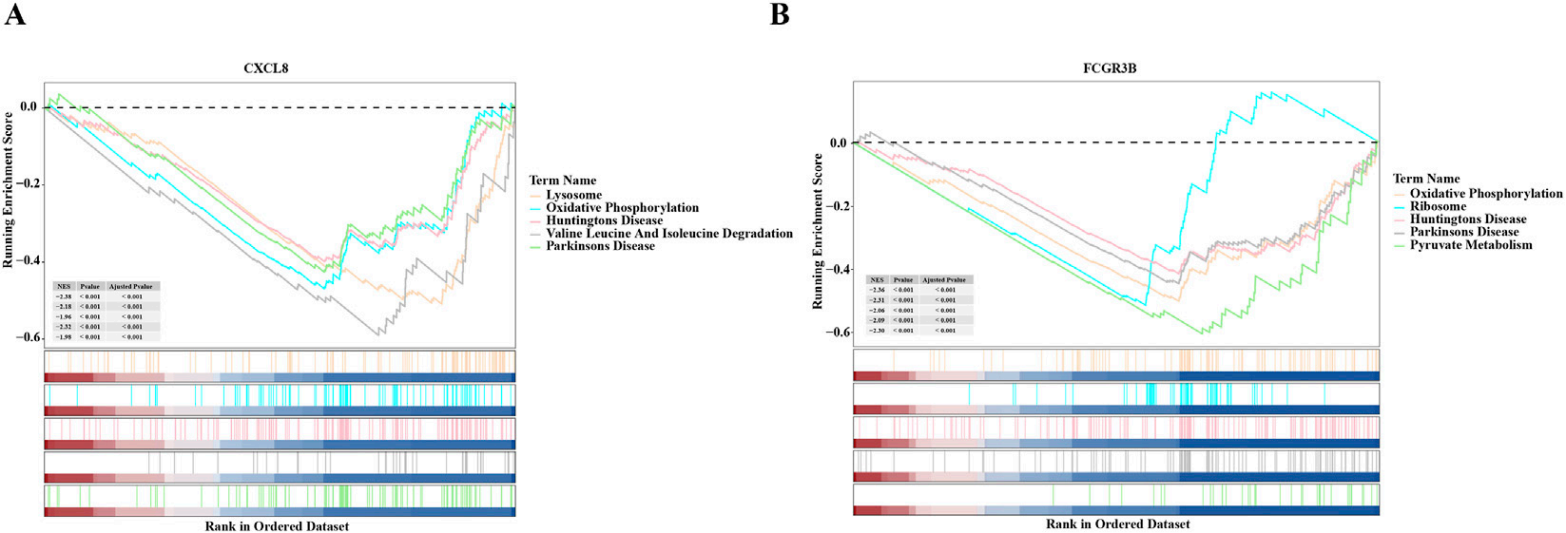
GSEA enrichment analysis of biomarkers. **(A)** Visualization of the top 5 most significant signaling pathways for CXCL8. **(B)** Visualization of the top 5 most significant signaling pathways for FCGR3B.

Further, GSEA was performed for HCHwCI and HCHsCI samples from transcriptome sequencing data (p < 0.05, |NES|>1), and CXCL8 was significantly enriched in 58 pathways (Supplementary Data Sheet S11). These pathways include Lysosome, Oxidative Phosphorylation, Huntington’s Disease, valine/leucine/isoleucine degradation, Parkinson’s Disease, and other pathways (Figure 6A). The FCGR3B gene was enriched in 60 pathways (Supplementary Data Sheet S12), and these pathways include Oxidative Phosphorylation, Ribosome, Huntington’s Disease, Parkinson’s Disease, Pyruvate Metabolism, and other pathways (Figure 6B). Based on these enrichment results, these results suggest that CXCL8 and FCGR3B may jointly affect Oxidative Phosphorylation, Huntington’s Disease, and Parkinson’s Disease, thereby participating in the occurrence and development of HCH.

### 3.7 The biological functions of 2 biomarkers were explored

**FIGURE 7:**
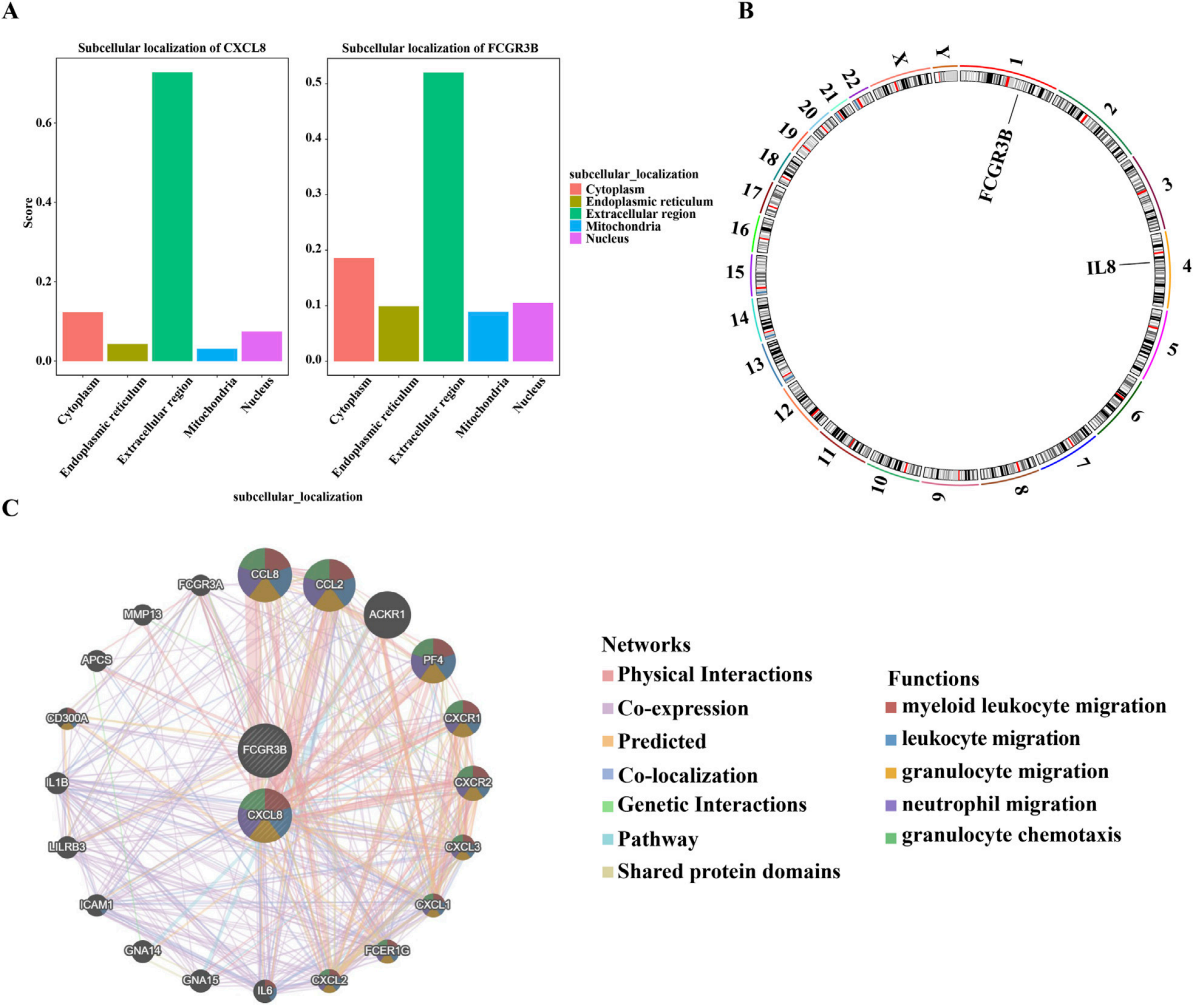
Subcellular localization, chromosomal localization, and GeneMANIA analysis of biomarkers. **(A)** Distribution of subcellular localization scores for CXCL8 (left) and FCGR3B (right). The horizontal axis represents subcellular localization, and the vertical axis represents the percentage of each biomarker distributed across five subcellular locations. Different colors indicate different subcellular compartments. Both are primarily localized in the extracellular region. **(B)** Chromosomal localization of the biomarkers: CXCL8 is located on chromosome 4, while FCGR3B is located on chromosome 1. **(C)** The biomarker interaction network shows 20 genes interacting with CXCL8 and FCGR3B, including myeloid leukocyte migration, leukocyte migration, granulocyte migration, neutrophil migration, and granulocyte chemotaxis.

Subcellular localization analysis of CXCL8 and FCGR3B genes was carried out. CXCL8 and FCGR3B genes were mainly distributed in the extracellular region (Figure 7A). This might imply that the proteins they encoded functioned primarily outside the cell. Chromosomal localization displayed that CXCL8 (IL8) was situated on chromosome 4, while FCGR3B lay on chromosome 1 (Figure 7B). GeneMANIA analysis identified 20 genes that interacted with CXCL8 and FCGR3B. These included CCL8, CCL2, ACKR1, PF4, CXCR1, CXCR2, CXCL3, CXCL1, FCER1G, CXCL2, IL6, GNA15, GNA14, ICAM1, LILRB3, IL1B, CD300A, APCS, MMP13, and FCGR3A (Figure 7C). Subsequently, CXCL8 and FCGR3B closely interacted in a variety of biological functions, which included myeloid leukocyte migration, granulocyte migration, and neutrophil migration. It can be presumed that CXCL8 and FCGR3B might be involved in the immune response process, thereby exerting an influence on HCHwCI.

### 3.8 Regulatory networks, drug and molecular docking of 2 biomarkers

**FIGURE 8:**
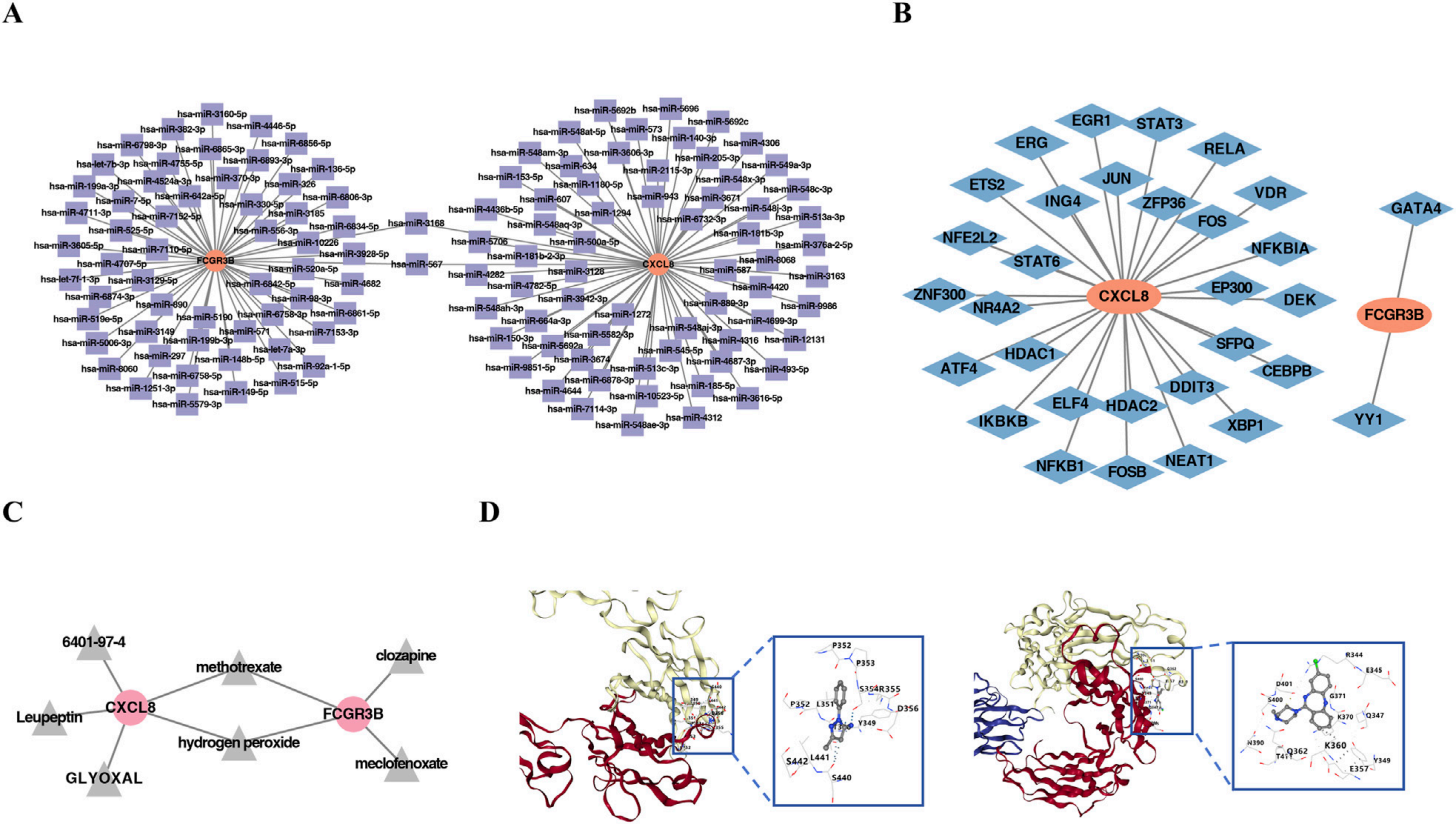
Molecular regulatory network analysis, drug prediction, and molecular docking. **(A)** Visualization of the biomarker-miRNA regulatory network, with 67 miRNAs regulating CXCL8 and 58 miRNAs regulating FCGR3B. **(B)** Visualization of the biomarker-transcription factor (TF) regulatory network, with 29 TFs targeting CXCL8 and 2 TFs targeting FCGR3B. **(C)** Drug-biomarker network, displaying the top 5 results with the highest relevance scores to the biomarkers. **(D)** Molecular docking models of CXCL8 (left) and FCGR3B (right) with their corresponding target drugs.

Using the database for miRNA prediction, a biomarker-miRNA regulatory network containing 125 key nodes and 125 interaction relationships was constructed. The network shows that 67 microRNAs (miRNAs) are predicted to regulate CXCL8, and 58 miRNAs are predicted to regulate FCGR3B (Supplementary Data Sheet S13). Notably, among these miRNAs, hsa-miR-3168 and hsa-miR-567 can regulate both genes simultaneously. They might have played an important regulatory role in HCHwCI (Figure 8A). Based on the prediction from the TRRUST database, a transcription factor-biomarker (mRNA) network was constructed. In this network diagram, there are 33 key nodes and 31 interactions. It was determined that there are 29 TFs targeting CXCL8 (such as CXCL8-STAT6) and 2 TFs targeting FCGR3B (such as FCGR3B-YY1) (Figure 8B; Supplementary Data Sheet S14).

In addition, DSigDB predicted 542 drugs that were associated with both biomarkers (Supplementary Data Sheet S15), including 540 drugs that target CXCL8, such as 6401-97-4, Leupeptin, and methotrexate. There were 4 drugs targeting FCGR3B. These drugs include clozapine and hydrogen peroxide (Figure 8C). Further molecular docking revealed that CXCL8 had a |Total Score| of 5.1 kcal/mol with 6401-97-4. Conformational binding analysis showed that multiple amino acid residues, such as P352, P353, and S354, stabilized the interaction. However, to our knowledge, this compound has not been characterized pharmacologically, and its clinical relevance remains unknown. Therefore, this finding should be viewed solely as a demonstration of the docking methodology rather than a genuine therapeutic lead. Similarly, FCGR3B had a |Total Score| of 6.3 kcal/mol with clozapine, with amino acid residues such as R344 and D401 contributing to the binding stability. However, clozapine is an antipsychotic medication with a black-box warning for agranulocytosis, and its approved indication is treatment-resistant schizophrenia, not cognitive impairment after stroke. Therefore, this result should also be interpreted as a demonstration of the docking methodology rather than a genuine therapeutic proposal for HCHwCI (Table 3; Figure 8D).

**TABLE 3:** List of total scores for biomarker - targeted drugs.

Key targets	Targeted drugs	Total score
CXCL8	6401-97-4	−5.1
FCGR3B	clozapine	−6.3

Important note: The drug prediction and molecular docking results presented here are derived from computational analyses and should be interpreted as hypothesis-generating rather than as therapeutic recommendations. All predicted compounds require extensive preclinical and clinical validation before any potential therapeutic application can be considered.

### 3.9 High expression of biomarkers in HCHwCI samples

**FIGURE 9:**
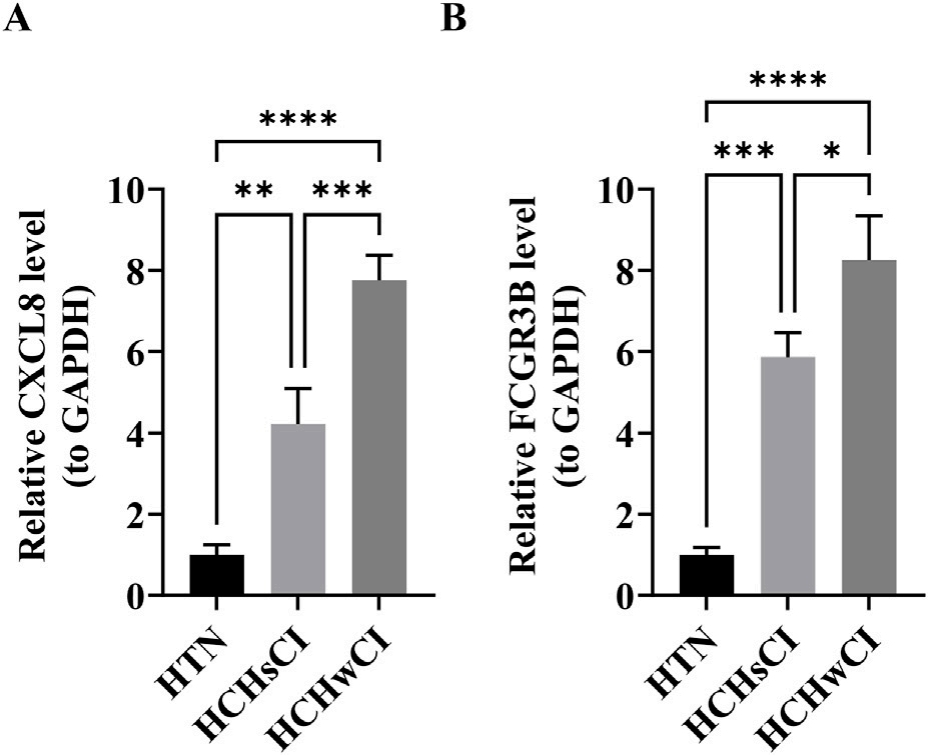
mRNA expression levels of two serum biomarkers in patients with HCHwCI and healthy controls, as determined by qRT-PCR. **(A)** Relative mRNA expression of CXCL8 normalized to GAPDH. **(B)** Relative mRNA expression of FCGR3B normalized to GAPDH. Data are presented as mean ± SEM. *p < 0.05, **p < 0.01 vs. control group (HTN).

Clinical samples were used for RT-qPCR validation of CXCL8 and FCGR3B. The results validated that CXCL8 and FCGR3B were significantly upregulated in HCHwCI samples (p < 0.05) (Figures 9A,B). Their expression patterns in clinical samples were consistent with the transcriptome sequencing data. In summary, the RT-qPCR validation of the biomarkers was consistent with the bioinformatics analysis and provided valuable insights into the gene expression patterns relevant to HCHwCI patients. This could support the advancement of personalized treatment approaches.

## 4 Discussion

HCHwCI is a severe neurological disorder triggered by hypertension. Although advances in surgical techniques and neurocritical care have reduced mortality rates, they have not effectively improved cognitive impairment among HCH survivors (Pasi et al., 2021). To address this clinical challenge, molecular-level investigation is essential. Transcriptome sequencing technology is an advanced RNA sequencing method used for whole-transcriptome analysis and differential gene expression interpretation. It has become an indispensable tool for transcriptomic analysis in examining gene expression and mRNA splicing variations (Stark et al., 2019). This study identified two biomarkers (CXCL8 and FCGR3B) through transcript sequencing, differential expression analysis, protein-protein interaction (PPI) screening, and expression level validation. Based on these biomarkers, gene set enrichment analysis, subcellular and chromosomal localization studies, and GeneMANIA analysis were performed to construct a molecular regulatory network. RT-qPCR validated the consistent expression of CXCL8 and FCGR3B in clinical blood samples, further exploring potential mechanisms and therapeutic targets in HCHwCI patients. Building on this pivotal role, we subsequently analyze the biological functions and clinical relevance of CXCL8 and FCGR3B.

CXCL8 (C-X-C motif chemokine ligand 8) is a potent neutrophil chemokine with minimal expression under normal physiological conditions. However, its expression can be swiftly upregulated by inflammatory mediators like tumor necrosis factor-α (TNF-α) or interleukin-1β (IL-1β), which then modulates cell proliferation and invasion through autocrine or paracrine signaling, contributing critically to immune responses during infection and tissue damage (Cambier et al., 2023; Xiao et al., 2025). Prior studies demonstrate a close association between CXCL8 and the pathology of ICH. Its expression in serum and cerebrospinal fluid is significantly upregulated following ICH, indicating that it may be a potential susceptibility gene for this disease (Seyfried et al., 2025; Rodney et al., 2018). In this study, RT-qPCR indicated heightened CXCL8 expression in HCHsCI and HCHwCI groups, with the HCHwCI group exhibiting a stronger increase, implying CXCL8’s involvement in post-HCH cognitive dysfunction. Further KEGG pathway enrichment analysis revealed significant enrichment of CXCL8 in pathways including “Chemokine Signaling” and “Cytokine-Cytokine Receptor Interactions”. Based on enrichment analysis results and considering the known function of CXCL8 in mediating neutrophil chemotaxis and activation through the chemokine signaling pathway, we propose the following hypothesis: Key inflammatory mediators such as TNF-α and IL-1β released from damaged brain tissue following ICH might potentially be associated with the upregulation of CXCL8 expression. Elevated CXCL8 levels could theoretically be correlated with the recruitment and activation of neutrophils to infiltrate the lesion site via the chemokine signaling pathway. Excessive accumulation and activation of neutrophils are generally thought to be linked to the release of reactive oxygen species and proteases, which might disrupt the blood-brain barrier and cause neuronal damage. This process may exacerbate local inflammatory cascades and cytokine network activation, potentially contributing to more severe neuroinflammation and secondary brain injury, thereby correlating with the onset and progression of HCHwCI. However, these mechanisms are currently speculative and require further *in vivo* and *in vitro* validation to establish any causal relationship.

FCGR3B (Fcγ receptor IIIb), also known as CD16b, belongs to the immunoglobulin superfamily (IgSF). This receptor is primarily expressed on the surface of neutrophils, serving as a molecular marker for these cells (Durand et al., 2001; Mysore et al., 2021). Its main function is to phagocytose small soluble immune complexes. Deficiency of this receptor may delay neutrophil clearance of immune complexes, thereby promoting a pro-inflammatory state. Additionally, FCGR3B binds to the Fc segment of IgG antibodies, triggering granulocyte release of inflammatory mediators such as reactive oxygen species and cytokines (Alyasova et al., 2022), thereby activating granulocyte functional activity. Studies indicate that copy number variations (CNVs) and single nucleotide polymorphisms (SNPs) in the FCGR3B gene correlate with various autoimmune and infectious diseases (Moraru et al., 2021). In granulomatous polyangiitis (GPA) and polyangiitis (MPA), elevated expression of FCGR3B on neutrophil surfaces may enhance anti-neutrophil cytoplasmic antibody (ANCA)-mediated neutrophil activation (Martorana et al., 2016). This promotes vasculitis and tissue damage and can predict the risk of GPA recurrence in MPA-associated cases. In patients with Rasmussen’s encephalitis (RE), rare deleterious variants in the FCGR3B gene may underlie clinical manifestations such as refractory epilepsy, progressive cognitive impairment, and motor decline (Leit et al., 2023). This study first identified abnormal FCGR3B expression in the blood of HCHwCI patients. Enrichment analysis suggests FCGR3B may participate in pathways including neutrophil degranulation and Fcγ receptor-mediated phagocytosis. Based on this, it is hypothesized that FCGR3B might be correlated with secondary neurological damage following intracerebral hemorrhage, possibly by interacting with the functional state of circulating neutrophils. This offers a new perspective for understanding the immunopathological mechanisms of this disease, although direct experimental evidence is needed to confirm this interaction.

To explore a wider molecular context, gene enrichment analysis was conducted, revealing that CXCL8 and FCGR3B were significantly enriched in pathways such as oxidative phosphorylation, Huntington’s disease (HD), and Parkinson’s disease (PD). This suggests their potential involvement in HCH pathogenesis through the synergistic regulation of energy metabolism, neurodegenerative processes, and inflammation. Recent research indicates that oxidative stress is a key driver of secondary brain injury, and as the central energy metabolism pathway, impaired oxidative phosphorylation can trigger oxidative stress and exacerbate neurological damage (Shao et al., 2022). Within the oxidative phosphorylation pathway, the altered expression of CXCL8 and FCGR3B might be associated with impaired mitochondrial function and enhanced reactive oxygen species accumulation post-hemorrhage, potentially exacerbating oxidative stress and neuronal injury (Fung et al., 2025). Among the HD pathways, it has been reported that significantly elevated central and peripheral IL-8 (CXCL8) levels in HD patients can exacerbate neuroinflammation by recruiting immune cells, whereas the enrichment of FCGR3B in this pathway suggests that it might theoretically be involved in antibody-dependent cytotoxicity. This could potentially amplify the immune response in concert with CXCL8, correlating with microglial cell activation and potential neuronal loss in HCH. In addition, the PD pathway has been shown to be closely associated with cognitive impairment and neuroinflammation, and CXCL8 has been widely reported to be a key mediator of neuroinflammation in PD, regulating immune cell infiltration and affecting dopaminergic neuron function through interactions with other chemokines (e.g., CCL2 and CCL5) (Wang et al., 2024), and the synergistic enrichment of FCGR3B further supports a role for the two in a similar inflammatory regulatory network Role. In summary, although the associations suggested by enrichment analyses originated from studies of other neurological disorders, it is likely that CXCL8 and FCGR3B play a synergistic role in the onset and progression of HCH by modulating the core pathological aspects (i.e., oxidative stress and neuroinflammation) that are shared by the above pathways.

Our regulatory network analysis identified hsa-miR-3168 and hsa-miR-567 as potential upstream regulators of both CXCL8 and FCGR3B. Among these, hsa-miR-567 has been previously implicated in mild cognitive impairment and vascular dementia (De Felice et al., 2020), providing indirect support for its potential role in HCHwCI. However, the functional significance of these miRNA-biomarker interactions in HCHwCI remains to be experimentally determined.

From a therapeutic perspective, computational drug prediction and molecular docking identified several compounds with predicted binding affinity to CXCL8 and FCGR3B, including clozapine and 6401-97-4. While these findings raise the possibility of targeting these biomarkers for therapeutic intervention, we emphasize that these are *in silico* predictions only. Extensive preclinical and clinical validation is required before any therapeutic applications can be considered.

Several limitations of this study must be explicitly acknowledged. Primarily, the sample sizes for both the transcriptome sequencing (n = 3 per group) and the subsequent RT-qPCR validation (total n = 10) are critically small. Such a limited cohort size intrinsically restricts the statistical power required for robust biomarker discovery and elevates the risk of statistical bias. Consequently, the current study should be viewed as preliminary and exploratory rather than conclusive. The identified roles of CXCL8 and FCGR3B serve as hypothesis-generating insights. To ensure the reliability, reproducibility, and clinical applicability of these findings, future research utilizing independent validation in larger, multi-center cohorts is absolutely imperative. Furthermore, the mechanistic pathways, including oxidative phosphorylation and neuroinflammation discussed herein, are derived extensively from bioinformatics predictions and literature correlations. The current study lacks *in vitro* or *in vivo* experimental evidence to validate these functional hypotheses. Therefore, the causal relationships between these biomarkers and the pathogenesis of HCHwCI remain to be experimentally determined. Furthermore, an exhaustive search of public repositories (e.g., the Gene Expression Omnibus database) yielded no suitable transcriptomic datasets matching our specific clinical criteria—namely, patients with hypertensive intracerebral hemorrhage meticulously grouped by cognitive impairment status. Consequently, *in silico* external validation utilizing independent public cohorts could not be performed at this stage. This data gap further underscores the indispensable need for our planned future multi-center, large-scale prospective cohort studies to independently validate the diagnostic value of CXCL8 and FCGR3B.

## 5 Conclusion

This exploratory study is the first to identify CXCL8 and FCGR3B as potential biomarkers for HCHwCI through integrated transcriptomic and bioinformatics approaches. The constructed molecular regulatory network and drug prediction model provide novel mechanistic insights and identify promising therapeutic targets for this condition. Furthermore, the consistent expression of CXCL8 and FCGR3B was verified by RT-qPCR in clinical blood samples, thereby providing a theoretical basis for early diagnosis and intervention in HCHwCI. In the future, further research will focus on elucidating the regulatory relationships among CXCL8, FCGR3B, and HCHwCI via carefully planned *in vivo* and *in vitro* experiments. Following validation in larger independent cohorts, this work will verify the reliability of our current results and contribute innovative directions to HCHwCI translation, spanning basic biomarker discovery to clinical diagnostic and therapeutic pathway refinement.

## Data Availability

The data presented in the study are deposited in the Zenodo repository, accession number https://doi.org/10.5281/zenodo.21203324.

## Supplementary material

SUPPLEMENTARY DATA SHEET S1 Primer sequences used for RT-qPCR. Contains the primer information referred to in the Methods section.

SUPPLEMENTARY DATA SHEET S2 Transcriptome sequencing quality metrics. Contains Q20, Q30, GC content and related raw sequencing quality information.

SUPPLEMENTARY DATA SHEET S3 DEGs1 list. Differentially expressed genes for HCHsCI vs. Control.

SUPPLEMENTARY DATA SHEET S4 DEGs2 list. Differentially expressed genes for HCHwCI vs. Control.

SUPPLEMENTARY DATA SHEET S5 DEGs3 list. Differentially expressed genes for HCHwCI vs. HCHsCI.

SUPPLEMENTARY DATA SHEET S6 GO enrichment results. Gene Ontology enrichment results for differential CRGs.

SUPPLEMENTARY DATA SHEET S7 KEGG enrichment results. KEGG enrichment results for differential CRGs.

SUPPLEMENTARY DATA SHEET S8 Degree algorithm hub genes. Top-ranked genes identified using the Degree algorithm.

SUPPLEMENTARY DATA SHEET S9 MCC algorithm hub genes. Top-ranked genes identified using the MCC algorithm.

SUPPLEMENTARY DATA SHEET S10 EPC algorithm hub genes. Top-ranked genes identified using the EPC algorithm.

SUPPLEMENTARY DATA SHEET S11 GSEA results for CXCL8. Significantly enriched pathways associated with CXCL8.

SUPPLEMENTARY DATA SHEET S12 GSEA results for FCGR3B. Significantly enriched pathways associated with FCGR3B.

SUPPLEMENTARY DATA SHEET S13 Predicted miRNA interactions. Predicted miRNAs regulating the biomarkers.

SUPPLEMENTARY DATA SHEET S14 Predicted transcription factors. Predicted transcription factors targeting the biomarkers.

SUPPLEMENTARY DATA SHEET S15 Predicted drug candidates. Drugs associated with the biomarkers based on DSigDB prediction.
